# Supplementary material for: Mammal dung–dung beetle trophic networks: an improved method based on gut-content DNA
Source: PeerJ. 2024 Mar 15;12:e16627. doi: 10.7717/peerj.16627 (PMC10946388; doi:10.7717/peerj.16627)
Supplement: Table S3 [file peerj-12-16627-s003.docx]

**Supplementary Table 3:**

From 18 paired sample results for 12s rDNA Primers

| Mammal | *Alouatta palliata* | *Ateles fusciceps* | *Cebus capucinus* | *Caluromys philander* |
| --- | --- | --- | --- | --- |
| Dung Beetle |  |  |  |  |
| *Canthon anagustatus* | 5 | 8 | 0 | 2 |
| *Oxysternon conspicullatum* | 1 | 0 | 0 | 0 |
| *Scybalocanthon trimaculatum* | 0 | 0 | 2 | 0 |
