# Supplementary material for: Mammal dung–dung beetle trophic networks: an improved method based on gut-content DNA
Source: PeerJ. 2024 Mar 15;12:e16627. doi: 10.7717/peerj.16627 (PMC10946388; doi:10.7717/peerj.16627)
Supplement: Table S4 [file peerj-12-16627-s004.docx]

**Supplementary Table 4:**

From 18 paired sample results for 16s rDNA Primers

| Mammals | *Alouatta palliata* | *Ateles fusciceps* | *Cebus capucinus* |
| --- | --- | --- | --- |
| Dung Beetle |  |  |  |
| *Canthon anagustatus* | 7 | 8 | 0 |
| *Oxysternon conspicullatum* | 1 | 0 | 0 |
| *Scybalocanthon trimaculatum* | 0 | 0 | 2 |
