# Supplementary material for: Mammal dung–dung beetle trophic networks: an improved method based on gut-content DNA
Source: PeerJ. 2024 Mar 15;12:e16627. doi: 10.7717/peerj.16627 (PMC10946388; doi:10.7717/peerj.16627)
Supplement: Table S5 [file peerj-12-16627-s005.docx]

**Supplementary Table 5:**

list of mammals from the German forest from 7

pitfall traps using only the MiMammal-U primers (Ushio et al. 2017)

| Mammal Species | Sum of beetles with iDNA of mammal species |
| --- | --- |
| *Apodemus flavicolli* | 12 |
| *Apodemus hermonensis* | 1 |
| *Capreolus capreolus* | 2 |
| *Garrulus glandarius* | 1 |
| *Homo sapiens* | 3 |
| *Myodes glareolus* | 1 |
| *Sciurus vulgaris* | 2 |
| *Sorex araneus* | 1 |
| Total | 23 |
