# Supplementary material for: Mammal dung–dung beetle trophic networks: an improved method based on gut-content DNA
Source: PeerJ. 2024 Mar 15;12:e16627. doi: 10.7717/peerj.16627 (PMC10946388; doi:10.7717/peerj.16627)
Supplement: Table S6 [file peerj-12-16627-s006.docx]

**Supplementary Table 6:**

Hand collected beetles Ecuador Forest 16 beetle hand collections

| Primer | 16smama (Taylor 1996) | MiMammal-U (Ushio et al. 2017) |
| --- | --- | --- |
| Mammal Species | |  |
| *Alouatta palliata* | 3 | 8 |
| *Ateles fusciceps* | 8 | 10 |
| *Cebus capucinus* | 2 | 3 |
| *Dasypus novemcinctus* | 0 | 1 |
| *Panthera onca* | 0 | 1 |
| *Puma concolor* | 0 | 5 |
| Total | 13 | 28 |
