# Supplementary figures and images for: Mammal dung–dung beetle trophic networks: an improved method based on gut-content DNA
Source: PeerJ. 2024 Mar 15;12:e16627. doi: 10.7717/peerj.16627 (PMC10946388; doi:10.7717/peerj.16627)

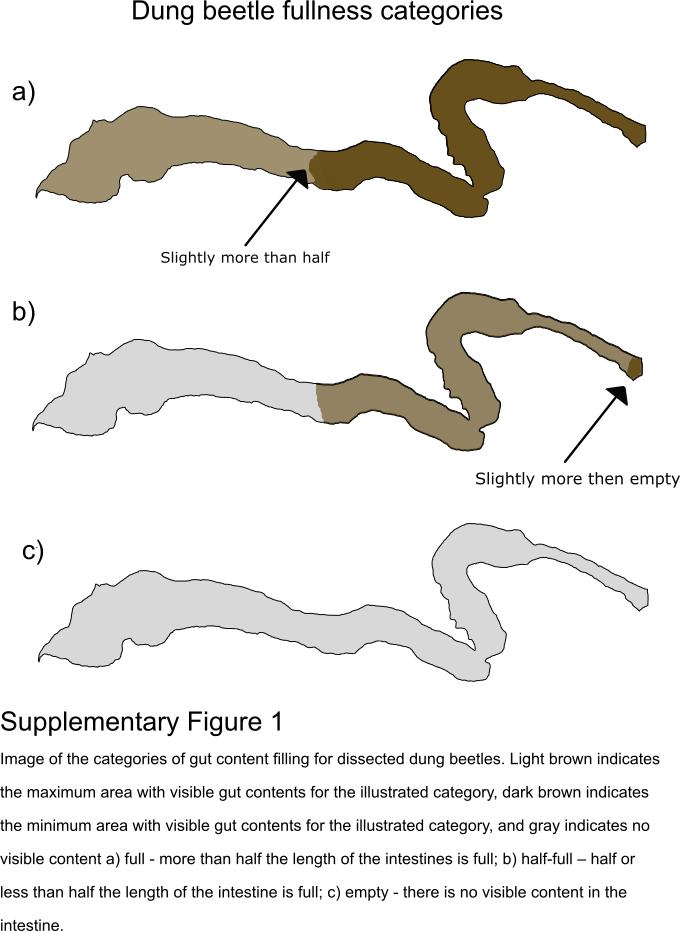

Supplement: Figure S1 — Light brown indicates the maximum area with visible gut contents for the illustrated category, dark brown indicates the minimum area with visible gut contents for the illustrated category, and gray indicates no visible content (A) full—more than half the length of the intestines is full; (B) half-full –half or less than half the length of the intestine is full; (C) empty—there is no visible content in the intestine. [file peerj-12-16627-s010.png]

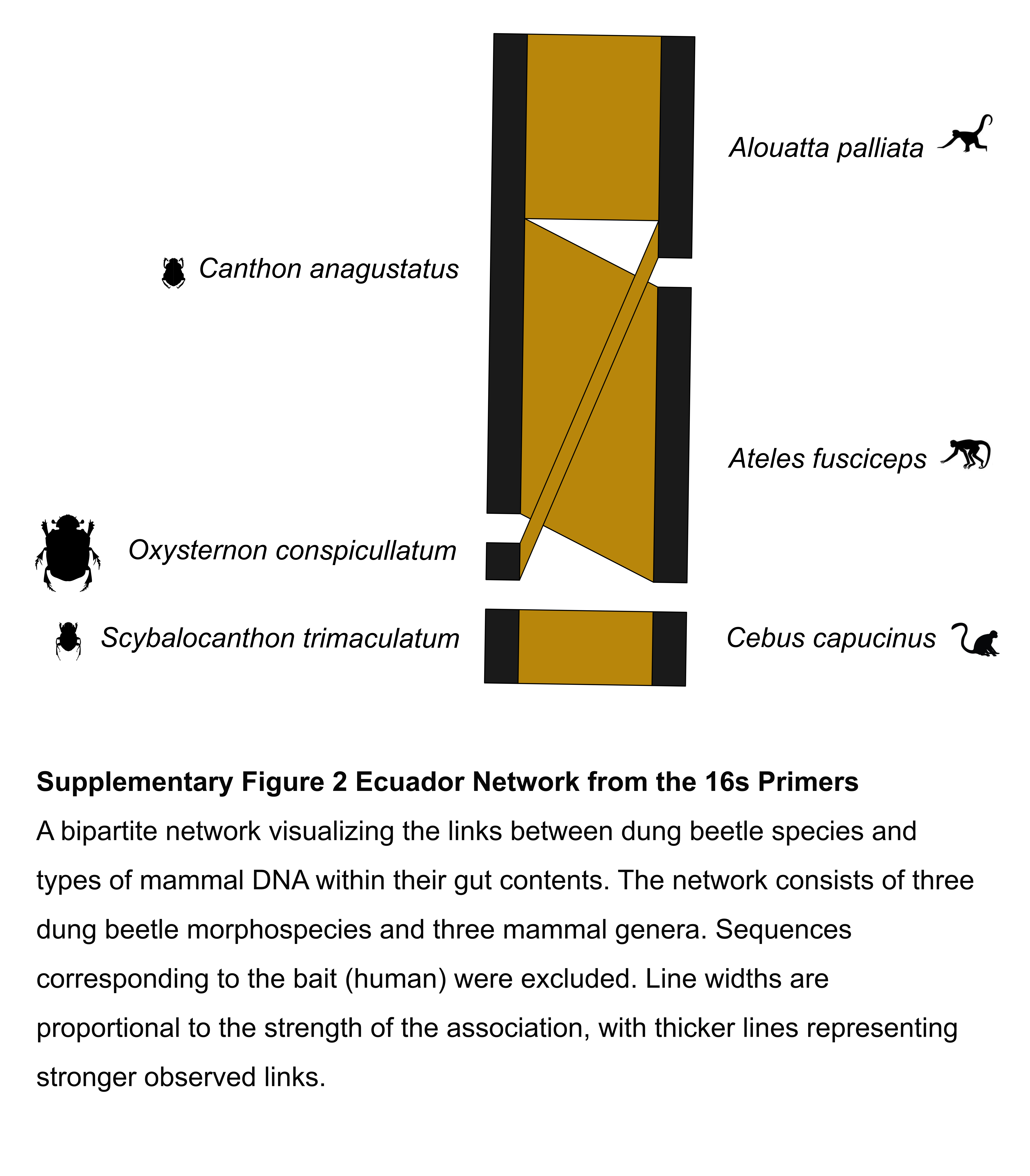

Supplement: Figure S2 — A bipartite network visualizing the links between dung beetle species and types of mammal DNA within their gut contents. The network consists of three dung beetle morphospecies and three mammal genera. Sequences corresponding to the bait (human) were excluded. Line widths are proportional to the strength of the association, with thicker lines representing stronger observed links. [file peerj-12-16627-s011.png]

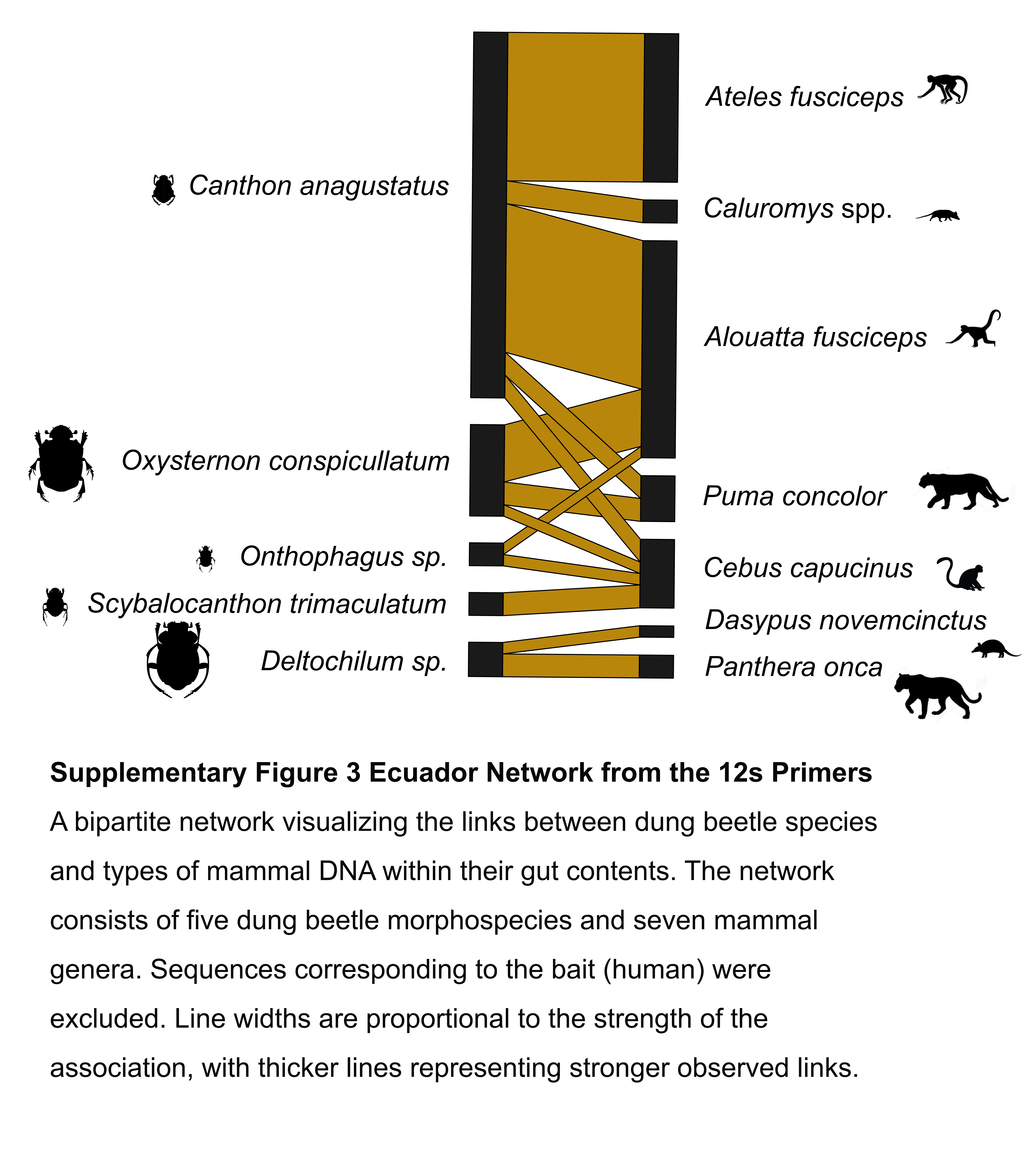

Supplement: Figure S3 — A bipartite network visualizing the links between dung beetle species and types of mammal DNA within their gut contents. The network consists of five dung beetle morphospecies and seven mammal genera. Sequences corresponding to the bait (human) were excluded. Line widths are proportional to the strength of the association, with thicker lines representing stronger observed links. [file peerj-12-16627-s012.png]
